# Draft Genome Sequences of *Ralstonia pickettii* Strains SSH4 and CW2, Isolated from Space Equipment

**DOI:** 10.1128/genomeA.00887-14

**Published:** 2014-09-04

**Authors:** Pieter Monsieurs, Kristel Mijnendonckx, Ann Provoost, Kasthuri Venkateswaran, C. Mark Ott, Natalie Leys, Rob Van Houdt

**Affiliations:** a Unit of Microbiology, Belgian Nuclear Research Centre (SCK•CEN), Mol, Belgium; b Biotechnology and Planetary Protection Group, Jet Propulsion Laboratory, California Institute of Technology, Pasadena, California, USA; c Biomedical Research and Environmental Science Division, NASA, Johnson Space Center, Houston, Texas, USA

## Abstract

*Ralstonia pickettii* SSH4 and CW2 were isolated from space equipment. Here, we report their draft genome sequences with the aim of gaining insight into their potential to adapt to these environments.

## GENOME ANNOUNCEMENT

*Ralstonia pickettii* SSH4 and CW2 are Gram-negative bacteria (*Burkholderiaceae* family) isolated pre-flight from the surface of the Mars Odyssey Orbiter during assembly at the Kennedy Space Center in Florida and from a water sample taken in-flight from the American segment of the International Space Station (ISS) cooling system, respectively (1–3).

*R. pickettii* strains are prevalent in water and soil (4). They have been recovered from many different water sources such as distilled water used in hospitals (5), dental unit water lines (6), public drinking water supplies (7), bottled water (8), ultrapure industrial water systems (9, 10), and even from drinking water systems of the Mir space station (11) and the Shuttle (12). *R. pickettii* has the ability to survive and thrive in oligotrophic conditions (4, 13) probably mediated by its biodegradative abilities (4), its large metabolic diversity, and its ability to form biofilms, making them more resistant to biocides and consequently more difficult to eradicate (14–16). In addition, *R. pickettii* has been recovered from a wide range of clinical environments and emerged as an opportunistic pathogen that should not be overlooked as a cause of nosocomial infections (17). The draft genome sequences reported here will help to elucidate how these strains are able to persist in these strictly controlled environments.

Whole-genome shotgun and paired-end sequencing of *R. pickettii* SSH4 and CW2 were performed by Macrogen (Seoul, Korea) using the 454 GS FLX sequencing platform. The sequencing data showed an average read length of 331 nt and 332 nt, an average insert size of 2877 nt and 2826 nt, and a total number of sequencing data of 367 Mbp and 337 Mbp for strains SSH4 and CW2, respectively. Both genomes were assembled using the Newbler software (version 2.3) resulting in 38 and 32 contigs and *N*_50_ values of 534,775 nt and 469,201 nt for SSH4 and CW2, respectively.

The genome of *R. pickettii* SSH4 was estimated to be 5,746,538 bp with a G+C content of 63.30%. The genome of *R. pickettii* CW2 was estimated to be 5,490,874 bp with a G+C content of 63.65%. Both strains harbor a chromosome, a chromid (18), and a megaplasmid (>230 kb). Strain SSH4 carries two additional plasmids of around 65 and 95 kb, respectively. These estimations were based on plasmid extraction and gel electrophoresis analysis as previously reported (1). The automated genome annotation of SSH4 through the MicroScope platform (19) identified 5,868 protein-coding genes of which 72.0% were classified in at least one cluster of orthologous group (COG), 49 tRNA genes, and 3 rRNA genes. The CW2 genome annotation displayed 5,598 protein-coding genes of which 73.7% were classified in at least one COG, 51 tRNA genes, and 3 rRNA genes. A general pan-genome analysis via the MicroScope platform (19) indicated that SSH4 and CW2 share 3,903 coding sequences.

### Nucleotide sequence accession numbers.

This whole-genome shotgun project has been deposited at DDBJ/EMBL/GenBank under the accession numbers JFZG00000000 and JFZH00000000 for strains SSH4 and CW2, respectively.
